# 

**Published:** 1904-09-03

**Authors:** 


**Notices of Births, Deaths, and Marriages** are inserted in this column at a charge of 2s. 6d. for an announcement not exceeding four lines.

### DEATH.

Cross.—On Aug. 22, Edith Eleanor Cross, of 30 Devereux Road, Wandsworth Common, formerly nurse at Bedford Infirmary and Netherfield Road Hospital, Liverpool. Aged 42. (2347)

### NOTICE TO CORRESPONDENTS.

All MSS., Letters, Books for Review, and other matters intended for the Editor should be addressed The Editor, "The Hospital," The Hospital Buildings, 28 & 29 Southampton Street, Strand, W.C.
The Editor cannot undertake to return rejected MS., even when accompanied by stamped directed envelope.

**Notice to Advertisers and Subscribers.**

All Advertisements, Orders for Copies of The Hospital, and Business Communications should be addressed to **THE MANAGER (Not to the Editor)**, The Hospital, 28 & 29 Southampton Street, Strand, London, W.C.
The Cost of Subscription, post free, is as follows:—Per week, 3½d.; per quarter, 3s. 3d.; per half-year, 6s. 6d.; per annum, 13s. Foreign and Colonial:—Per annum, 19s. 6d., payable, in all cases, in advance.

**NOTICE.—Vol. XXXV. of "The Hospital" (October 1903 to March 1904), in handsome cloth binding, is now on sale at the office of this Journal, price 10s. 6d. Cases for binding the Numbers contained in this Volume 2s. Index to Vol. XXXV., 3½d., post free.**
**The Monthly part for September is now ready. Price 1s., by post, 1s. 3d.**

### Medical Appointments.

Henry Hanna, M.A., B.Sc., M.B., R.U.I., Demonstrator in Anatomy, Queen's College, Belfast. N. R. Haswell, M.R.C.S.Eng., Medical Officer to the Helston Rural District Council. W. S. Kerr, M.B., C.M.Edin., F.R.C.S.Edin., Honorary Surgeon to Ear and Throat Department, Sheffield Royal Infirmary. W. W. Leigh, M.R.C.S.Eng., L.R.C.P.Edin., Medical Officer of Llanfabon. D. S. Park, F.R.C.S.E., Medical Officer to the Houghton-le-Spring Union. A. T. Spanton, M.A.Camb., L.S.A., House-Surgeon to the Hulme Dispensary, Manchester.

**"The Hospital" Institutional Library.**

**"Hospitals and Asylums of the World." 4 Vols., with a Portfolio of Plans, complete £12 12s.**

**"Burdett's Hospitals and Charities, 1904." 5s. net.**

**"Burdett's Official Nursing Directory." 8s. net.**

**"Cottage Hospitals, General, Fever, and Convalescent." 10s. 6d.**

**"Uniform System of Accounts for Hospitals and Public Institutions." New Edition. 4s. net.**

**"Hospital Expenditure: The Commissariat." 2s. 6d.**

**"A Legal Handbook for the Use of Hospital Authorities." 2s. 6d.**

**"The Cottage Hospital Case Book or Register of Patients." 10s. and 12s. 6d.**

**All these are published by the Scientific Press, Ltd., and may be obtained through any bookseller, or direct from the publishers, 28 and 29 Southampton Street, Strand, London, W.C.**

### BOOKS FOR DISTRICT NURSING ASSOCIATIONS.

### Hints on How to Start a District Nursing Association.

By a COUNTY SUPERINTENDENT.

This work, which is the outcome of great personal experience, gives full particulars on How to Start a District Nursing Association, and will be found of the greatest value to those who desire to establish such an Institution in their own immediate neighbourhood.

*Price* **1/-** *net;* **1/1** *post free.*

**NURSES' BOOKS** designed in accordance with the system contained in "Hints on How to Start a District Nursing Association."

**VISIT BOOK.** 2/- net; 2/3 post free.
**TIME BOOK.** 1/6 net; 1/7 post free.
**LOAN BOOK.** 1/- net; 1/1 post free.
**"SIMPLEX" CASE-BOOK.** 6d. net; 7d. post free.

Ruled and printed on good paper for the practical use of the District Nurse. Uniformly bound in Green Cloth.

Complete Catalogue of Nursing Manuals Post Free on Application.

*The above Works can be obtained of all Booksellers, or of*
THE SCIENTIFIC PRESS, LTD., 28 & 29 Southampton St., Strand, London, W. C.

